# Research of the Abnormal Mechanism of Brain Functional Network in Early Mild Cognitive Impairment Patients

**DOI:** 10.1049/syb2.70063

**Published:** 2026-04-15

**Authors:** Dong Qi, Weiping Wang, Yapeng Diao, Wenxiu Zhao, Wenqin Rong, Haiyan Zhao

**Affiliations:** ^1^ School of Computer and Communication Engineering University of Science and Technology Beijing Beijing China; ^2^ China United Network Communications Corporation Shandong Branch Jinan China; ^3^ Shunde Innovation School University of Science and Technology Beijing Shunde China; ^4^ Department of Neurology Peking University Third Hospital Beijing China

**Keywords:** biocomputers, brain models, feature extraction, feature selection

## Abstract

At present, the brain neural mechanism of the onset of early mild cognitive impairment (EMCI) patients has not yet been clarified, and it is difficult to detect the pre‐disease stage of EMCI patients and to carry out the subsequent intervention treatment. In this paper, by constructing the brain functional networks of the two subject groups of EMCI and Normal Control (NC), the mechanism of abnormalities in the brain functional networks and brain regions of the patients was investigated. Using the analysis method of complex network combined with graph theory, the global topological metrics of the brain functional networks of the subjects were investigated. Considering the compensatory mechanism of brain regions, this study starts with more fine‐grained brain regions to analyse the differential characteristics of brain functional networks between patients and normal subjects. Finally, we draw the preliminary conclusion that the global structure of the brain functional network of EMCI patients has been fundamentally reorganised, and the working mechanism has been abnormally changed, and we have unearthed the abnormal brain regions that cause the global changes of the brain functional network.

## Graphical abstract

This study constructed brain functional networks for early mild cognitive impairment (EMCI) patients and normal control (NC) groups, employing complex network and graph theory analysis to investigate the abnormal mechanisms in brain functional networks and regions of EMCI patients. The findings revealed that, although the global topological properties of the brain functional networks in EMCI patients may not show significant differences, a finer‐grained analysis of brain regions identified key abnormal regions driving global changes in the network. The preliminary conclusion suggests that the global structure of the brain functional network in EMCI patients has undergone fundamental reorganization, with abnormal alterations in its working mechanisms.

## 1 Introduction

Alzheimer's disease (AD) is a common neurodegenerative disease that usually manifests itself as brain nerve cell damage and memory loss, and is irreversible, making it difficult to treat and cure once it develops [1, 2]. Currently, there are more than 50 million Alzheimer's disease patients in the world [3], and the daily life of these patients is seriously disturbed by the disease [4, 5], so it is crucial to find ways to screen and cure Alzheimer's disease, and to intervene early and treat the patients before the onset of the disease.

Mild cognitive impairment (MCI) is considered a prodromal stage of Alzheimer's disease [6, 7]. Approximately 10%–20% of MCI patients progress to Alzheimer's disease each year, a rate significantly higher than that of cognitively normal elderly individuals [8]. With appropriate cognitive interventions and treatments, MCI patients have a high probability of slowing disease progression and may even experience a return to a healthy cognitive state [9].

MCI can be further categorised into early MCI (EMCI) and late MCI (LMCI) [10]. Early detection of EMCI patients, understanding the pathogenesis of EMCI, and intervening with appropriate treatments could significantly control the progression of Alzheimer's disease and alleviate patients' suffering [11]. However, the underlying brain mechanisms of EMCI remain unclear, making early screening and subsequent treatment challenging. Therefore, in‐depth research into the pathogenesis of EMCI and the identification of effective interventions are crucial.

Currently, the screening and diagnosis of early mild cognitive impairment mainly rely on cerebrospinal fluid index test [12] brain image analysis combined with the patient's mental state psychiatric scale assessment [13, 14] and other means, this process needs to rely on the professional doctors in large medical institutions to complete, and the simple examination methods cannot be completely accurate diagnosis of the onset of the patient's condition. Therefore, this has led to many patients with mild cognitive impairment missing the best period of disease detection and treatment. At the same time, cerebrospinal fluid index testing methods will cause some trauma to the patient's body when extracting cerebrospinal fluid from the patient [15]. With the rapid development of non‐invasive neuroimaging technology, more and more people have begun to pay attention to the neuroimaging technology to analyse the neural activity changes in the brain, so as to analyse the pathogenesis of neurodegenerative diseases [16].

Magnetic Resonance Imaging (MRI, Magnetic Resonance Imaging) is a medical imaging technique that is non‐invasive to the human body. This technique generates high‐resolution images, which can clearly show the internal structure of each organ of the human body, and is particularly suitable for imaging soft tissues, such as the brain, the spinal cord, the muscles, and the internal organs, etc. [17, 18, 19]. Functional Magnetic Resonance Imaging (fMRI) is a method that utilises MRI technology to measure and map the functional activity of the brain. fMRI focuses on detecting changes in the functioning of brain regions, mapping the areas of the brain that are active when performing specific cognitive or motor tasks [20, 21].

Brain functional network is a kind of network model that can reflect the neurosexual activity of the brain, which can be constructed through magnetic resonance imaging technology combined with complex network theory [22]. The analysis based on brain functional network can dig out the pathogenesis and specific indexes of EMCI patients, realize the early screening and late intervention treatment of EMCI patients, delay the onset of AD, and reduce the pain of patients.

**FIGURE 1:**
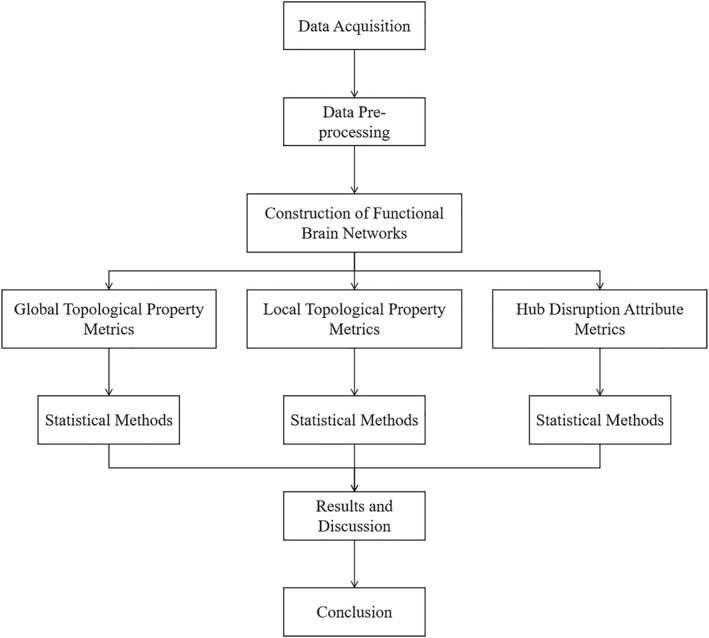
An overall flow chart.

The overall pipeline of this study is shown in Figure 1. This study establishes an innovative computational framework for investigating early mild cognitive impairment through three key advancements:

- We develop a multi‐scale analytical approach combining global topological metrics and regional hub disruption patterns to reveal compensatory mechanisms in brain network reorganization;
- We identify specific pathological reorganization signatures characterised by decreased network integration and altered nodal centrality as potential diagnostic biomarkers;
- Our designed metrics demonstrate superior detection sensitivity for subtle network abnormalities across diverse clinical cohorts.

## 2 Information and Methods

### 2.1 Data Acquisition

Functional MRI data of EMCI patients and normal subjects were obtained by downloading from ADNI's dataset (https://adni.loni.usc.edu/) [23], including data from 45 EMCI patients (EMCI group) and 45 normal subjects (NC group), and the demographics of the subjects are shown in Table 1. The subjects' MRI data were all acquired on a 3.0T Philips MRI machine. fMRI was performed using a planar echo sequence, and the acquisition sequence for each subject contained 197 time points, each consisting of 48 layers of slices with a size of 64 × 64 mm^2^, layer thickness of 3.3 mm, voxel size of 3.31 × 3.31 × 3.31 mm^3^, and a field of view of 220 × 220 mm^2^, with an echo time TE = 30 ms, repetition time TR = 3000 ms, and deflection angle FA = 90°.

**TABLE 1:** Demographic information of the study subjects.

Sample category	NC	EMCI
Number	45	45
Age (mean ± SD)	72.2 ± 6.1	73.6 ± 6.3
Male/Female	22/23	24/21
Mini‐mental state examination (MMSE) score (mean ± SD)	28.9 ± 1.3	27.7 ± 1.6
Clinical dementia rating (CDR) score	0	0.5

### 2.2 Data Pre‐Processing

MRI data were preprocessed by DPABI toolbox (http://rfmri.org/DPABI) [24], with the following main steps: (1) removal of the first 10 time points, in order to retain stable signals; (2) temporal layer correction to ensure that all scanning layers are at the same time point; (3) head movement correction to remove head movement interference; (4) alignment and segmentation; (5) spatial normalisation, using EPI templates to map brain imaging data (both T1‐weighted images and fMRI) to the common MNI (Montreal Neurological Institute) standard space; (6) spatial smoothing, to reduce noise and improve signal‐to‐noise ratios; (7) denormalisation, to remove the of linear trends; (8) filtering, to adjust the signal frequency characteristics, the filtering range chosen here was 0.01–0.1 Hz; (9) removal of other irrelevant signals such as head movement heartbeats; and (10) regression analysis, to regress the cerebrospinal fluid and cerebral white matter signal components.

### 2.3 Construction of Functional Brain Networks

Based on the AAL (Automated Anatomical Labelling) brain template [25], the brain was divided into 90 brain regions, and each brain region was used as a node of the brain functional network, and the corresponding BOLD (Blood Oxygen Level Dependent) signal time series of each brain region was extracted based on the preprocessing of the fMRI data. The average signal of all voxels in each brain region was used as the signal time series of the brain region; the Pearson correlation coefficients of the BOLD signal time series between two brain regions were calculated as the functional connection, and the brain functional network connection matrix was obtained. Since there is no clear conclusion about the biological significance of negative brain functional connectivity, only positive connectivity was retained in the brain functional network connectivity matrix, and the negative connectivity was set to 0, whereas the autoconnectivity was also set to 0. In order to make the data distribution of the matrix normal, the Fisher‐z method was used to further standardise the functional matrix with a z‐score. In order to further remove the noise from the data and remove the possible false connections in the functional connectivity matrix with weak connections and retain only the stronger connections, the functional connectivity matrix needs to be binarised. In this paper, the sparsity threshold method is used for binarisation, and the sparsity is selected to be 0.3 (i.e., retaining the functional connections with the strength of the top 30%), and in order to ensure the stability of the analytical results, two additional sparsities, 0.2 and 0.4, will be selected for comparison and verification.

### 2.4 Global Topological Property Metrics for Brain Functional Networks

The characteristic path length represents the average shortest path length between all nodes in the network. The formula for calculating the characteristic path length is as follows:

(1) $$L=\frac{1}{N\left(N-1\right)}\sum_{X,Y\in N,X\neq Y}l\left(X,Y\right).$$

In Equation (1), *N* is the number of brain region nodes in the brain functional network, and *l* (*X*, *Y*) is the shortest path from brain region *X* to brain region Y in the brain functional network, which is defined here as the minimum number of connected edges from *X* to Y.

The global efficiency characterisation measures the overall connectivity efficiency of the network. The global efficiency is calculated as follows:

(2) $$E_{glob}=\frac{1}{N\left(N-1\right)}\sum_{X,Y\in N,X\neq Y}\frac{1}{l\left(X,Y\right)}.$$

In Equation (2), *N* is the number of brain region nodes in the brain functional network, and *l* (*X*, *Y*) is the shortest path length from brain region *X* to brain region Y in the brain functional network, which is defined here as the minimum number of connections from *X* to Y.

The clustering coefficient characterises the degree of aggregation of nodes in a functional brain network. The clustering coefficient is calculated as follows:

(3) $$C_{X}=\frac{2E_{X}}{D_{X}\left(D_{X}-1\right)}.$$

(4) $$C=\frac{1}{N}\sum_{X=1}^{N}C_{X}.$$

In Equations (3) and (4), *N* is the number of brain area nodes in the brain functional network, *E*_*X*_ is the number of edges that actually exist between neighbouring nodes of brain area node *X*, and *D*_*X*_ is the node degree centrality of brain area node *X*.

### 2.5 Local Topological Property Metrics for Functional Brain Networks

Degree centrality measures how well a brain region node is connected in a functional brain network. The formula for calculating degree centrality is as follows:

(5) $$D_{X}=\sum_{X,Y\in N,X\neq Y}A_{XY}.$$

In Equation (5), *N* is the number of brain area nodes in the brain functional network and *A*_*XY*_ is an element in the brain functional network connection matrix, *A*_*XY*_ *=* 1 if there is a functional connection connected between brain area node *X* and brain area node *Y*, otherwise *A*_*XY*_ *=* 0.

Nodal median centrality represents the proportion of a brain node among all the shortest paths in a functional brain network, reflecting the role of a brain node as a bridge between other brain nodes in the network. The formula for median centrality is as follows:

(6) $$B_{X}=\sum_{\begin{array}{l} X,Y,Z\in N,\\ Y\neq X\neq Z \end{array}}\frac{\sigma_{YZ}\left(X\right)}{\sigma_{YZ}}.$$

In Equation (6), *σ*_*YZ*_ is the total number of shortest paths from brain region node *Y* to brain region node *Z*, and *σ*_*YZ*_ *(X)* is the number of shortest paths from brain region node *Y* to brain region node *Z* through *X*.

Local efficiency is a measure of connectivity between a brain region node and its neighbouring brain region nodes in a brain functional network, reflecting local connectivity and fault tolerance in the brain functional network. The formula for local efficiency is as follows:

(7) $$E_{\text{loc}}\left(X\right)=\frac{1}{N_{X}\left(N_{X}-1\right)}\sum_{Y,Z\in G_{X},Y\neq Z}\frac{1}{l\left(Y,Z\right)}.$$

In Equation (7), *N*_*X*_ is the number of neighbours of brain region node *X*, *G*_*X*_ denotes the set consisting of neighbours of brain region node *X*, and *l* (*Y*, *Z*) denotes the length of the shortest path between neighbouring nodes *Y* and *Z*. Only the shortest paths mediated through the neighbours of brain region node *X* are calculated.

### 2.6 Hub Disruption Attribute Metrics

The Hub Disruption Index (HDI) is a global metric that can be used to reflect the integrity of a functional brain network by measuring the disruption of brain region nodes as hubs in the network, and is sensitive to differences in the nodes in the network. For a given measure of the topological properties of a functional brain network, it can be further measured by comparing the degree of difference between the group mean value of each brain region node in EMCI patients and that of each brain region node in normal subjects.

**FIGURE 2:**
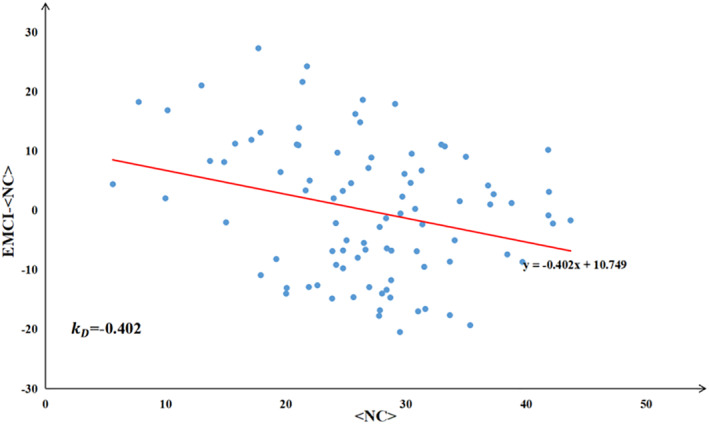
Degree centrality pivot disruption index plot for a single EMCI subject, with the *x*‐axis being the mean degree centrality of each brain region node group in NC subjects, and the *y*‐axis being the difference between the degree centrality of each brain region node in EMCI subjects and the mean degree centrality of each brain region node group in NC subjects.

The pivot interruption index was defined as a k‐value, which was obtained by calculating the difference between the functional brain network brain area node metrics of each EMCI subject and the mean value of the functional brain network brain area node metrics of the NC subject group, and further obtaining a fitted straight line by linearly fitting the data of these differences, and calculating the gradient value of the fitted straight line; each metric pivot interruption index was obtained by calculating it based on the local network topology attribute metrics. The linear fitting method used in this study is the least squares method, whose main idea is to find the parameters of the best‐fit model by minimising the sum of squares of the residuals (differences) between the data points and the fitted model. The solution of the least squares method has a good optimality property, which means that the fitting result is somehow the most reasonable. For linear models, the solution of the least squares method is unique, that is, there exists and is uniquely determined a straight line such that the data minimise the sum of the squares of the residuals with respect to that line. The pivot break index *k*_*D*_ for degree centrality for a single EMCI subject is shown in Figure 2.

### 2.7 Statistical Methods

Based on the computationally obtained global topological metrics and hub interruption attribute metrics of the brain functional networks of NC and EMCI subjects, this paper used SPSS to perform statistical analysis of variance. The topological metric measures and hub interruption attribute measures of brain functional networks were first tested for normal distribution using the method of descriptive statistical analysis in SPSS, and the results of the topological attribute measures and hub interruption attribute measures that satisfy normal distribution were analysed and compared by using independent samples t‐tests, whereas the results of the topological measures that do not satisfy normal distribution were analysed and compared by using the non‐parametric test Mann‐Whitney U‐tests. The results were analysed and compared, and the significance *p*‐value was statistically significant at less than 0.05 and strongly statistically significant at less than 0.01.

## 3 Results and Discussion

### 3.1 Analysis of Global Topological Property Metrics for Functional Brain Networks

**FIGURE 3:**
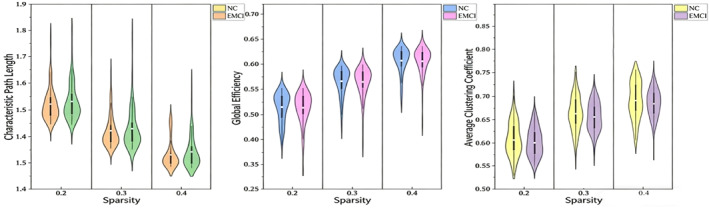
Statistical analysis violin plots of characteristic path length (left), global efficiency (middle), and clustering coefficient (right) of brain functional networks in EMCI and NC groups at sparsity of 0.2, 0.3, and 0.4.

In this paper, we chose the binarised brain functional networks at three sparsities of 0.20, 0.30, and 0.40, and statistically analysed the three network topological metrics, namely, characteristic path lengths, global efficiency and global average clustering coefficient, of the brain functional networks of the two subject groups of NC and EMCI, in order to improve the stability and reliability of the statistical analyses, and the results are shown in Figure 3. Measurements and statistics of global topological metrics of brain functional networks at different sparsities revealed that the group means of characteristic path length of brain functional networks of EMCI patients were all greater than those of NC subjects, and the group means of global efficiency and clustering coefficients were smaller than those of NC subjects. The characteristic path length of the EMCI group was 1.6 ± 0.2, and that of the NC group was 1.65 ± 0.25. There were no significant differences between the two groups in terms of characteristic path length, overall efficiency, and global average clustering coefficient after statistical analysis (*p* < 0.05).

Despite the lack of statistical significance, our findings suggest a trend that may indicate subtle changes in the global structure of brain functional networks in EMCI patients. These changes could be related to the early pathological processes of EMCI and warrant further investigation in future studies. Our results highlight the importance of considering different sparsity levels when analysing brain functional networks and suggest that more sensitive metrics may be needed for the early detection of EMCI.

### 3.2 Analysis of Interruption Mechanisms of Brain Functional Network Hubs

**FIGURE 4:**
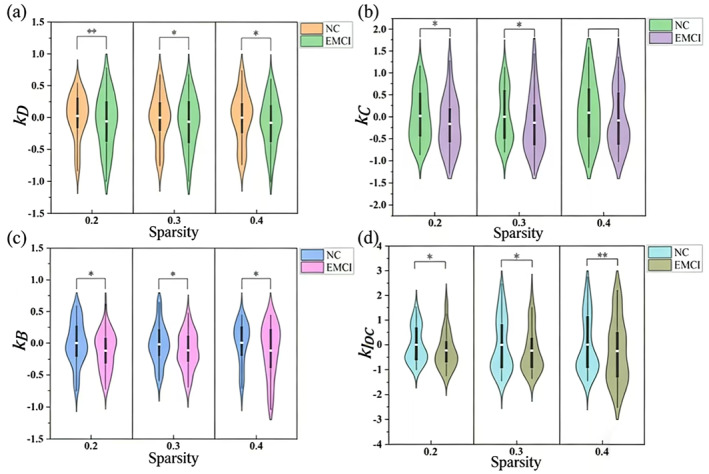
Violin plots of the statistical analysis of pivot disruption indices of brain functional networks in the EMCI and NC groups at sparsity levels of 0.2, 0.3, and 0.4; (a) degree centrality pivot disruption index; (b) clustering coefficient pivot disruption index; (c) median centrality pivot disruption index; and (d) local efficiency pivot disruption index.

Based on the local topological properties of the brain functional network mentioned earlier, the pivotal interruption indices of node degree centrality, node clustering coefficient, node median centrality, and node local efficiency of the brain functional network for each subject were obtained in this paper by calculating the slopes of the regression lines between the group averages of the local topological properties of the brain region nodes of the subjects in the NC group and the differences between the subjects in the NC group and the subjects in the EMCI group, respectively, and were denoted as *k*_*D*_, *k*_*C*_, *k*_*B*_, *k*_loc,_ respectively. The statistical analysis of the pivotal disruption indices of the functional brain networks of the EMCI group and the NC group at a sparsity of 0.2, 0.3, and 0.4 is shown in Figure 4.

**FIGURE 5:**
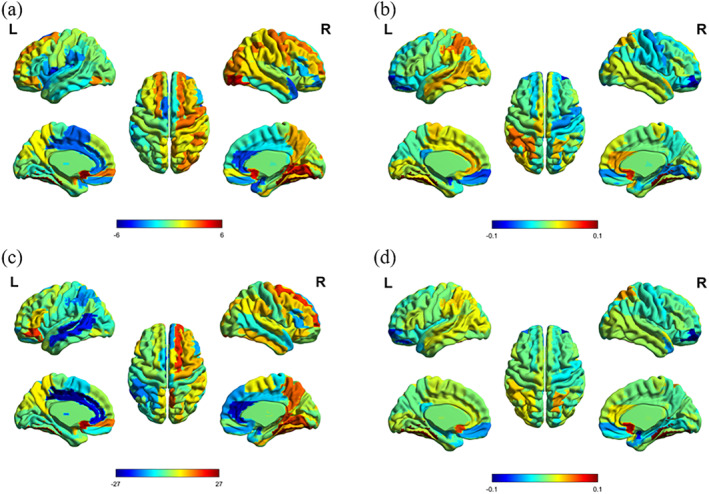
Group‐level average node degree centrality (a), clustering coefficient (b), median centrality (c), and local efficiency (d) of 90 brain regions of subjects in the EMCI group compared with the group‐level average abnormal brain region map of subjects in the NC group.

The study of pivot disruption index based on group's degree centrality (Figure 4a), clustering coefficient (Figure 4b), meso centrality (Figure 4c), and local efficiency (Figure 5d) found that *k*_*D*_, *k*_*C*_, *k*_*B*_, and kloc group means of the EMCI group were significantly negative and lower than those of the NC group. After statistical analysis, *k*_*D*_, *k*_*C*_, *k*_*B*_, *k*_loc_ were found to be significant (*p* < 0.05) between the two groups. This indicates that EMCI patients showed abnormally elevated or reduced brain functional network topology properties compared with normal individuals. For example, compared with normal human brain functional networks, for low degree centrality nodes, EMCI patients showed an increase, whereas for high degree centrality nodes, EMCI patients showed a general decrease; compared with normal human brain functional networks, for low clustering coefficients nodes, EMCI patients showed an increase, whereas for high clustering coefficients nodes, EMCI patients showed a general decrease. Showed a generalised decrease. This reflects a fundamental reorganization of the functional brain network in EMCI patients.

### 3.3 Analysis of Abnormal Brain Regions for Hub Disruption

Further, in this paper, we investigated the abnormal brain regions of pivotal disruption in EMCI patients, and here, group‐level averages of each brain region in EMCI subjects were used for comparative analyses with group‐level averages of each brain region in NC subjects, as shown in Figure 5.

As shown in Figure 5a, in the EMCI group, the degree centrality of the left olfactory cortex (OLF.L), right olfactory cortex (OLF.R), right inferior occipital gyrus (IOG.R), and right lingual gyrus (LING.R) was abnormally elevated to a greater extent, and the degree centrality of the left central sulcus (ROL.L), right nucleus of the sulcus cereus (PUT.R), and the left pulvinar alveolar bulb (PAL.L) was abnormally reduced to a greater extent. Showed a large degree of abnormal reduction. As shown in Figure 5b, the clustering coefficients of the right olfactory cortex (OLF.R), the left inferior parietal marginal angular gyrus (IPL.L), and the left superior parietal gyrus (IPL.L) were abnormally elevated, and the clustering coefficients of the left superior orbital frontal gyrus (ORBsup.L), the right middle orbital frontal gyrus (ORBmid.R), and the right pulsatile alveolus (PAL.R) were abnormally lowered in EMCI group. A large degree of abnormal decrease. As shown in Figure 5c, the EMCI group showed a large degree of abnormal elevation of median centrality in 4 brain regions, the left olfactory cortex (OLF.L), the right dorsolateral superior frontal gyrus (SFGdor.R), the left orbitoinferior frontal gyrus (ORBinf.L), and the right lingual gyrus (LING.R), and a large degree of abnormal elevation of median centrality in the left anterior and paracortical cingulate brain gyrus (ACG.L), and in the right anterior and paracortical cingulate brain gyrus (ACG.R), 4 brain regions, the left medial and paracentral cingulate gyrus (DCG.L) and the left middle temporal gyrus (MTG.L), showed a large degree of abnormal decrease in median centrality. As shown in Figure 5d, the EMCI group showed a greater degree of abnormal increase in the local efficiency of 3 brain regions, the left olfactory cortex (OLF.L), the right olfactory cortex (OLF.R), and the right supraparietal gyrus (SPG.R), and a greater degree of abnormal decrease in the local efficiency of the right orbital mid‐brain frontal gyrus (ORBmid.R).

As shown in Figure 5a,d, the concurrent increases in degree centrality (Figure 4a) and local efficiency (Figure 4d) in bilateral olfactory cortices (OLF.L/R) may indicate compensatory reorganization of olfactory systems responding to early neurodegeneration, with this pattern aligning with clinical manifestations of olfactory dysfunction in prodromal Alzheimer's disease and suggesting their biomarker potential; as shown in Figure 5b,d, significant reductions in clustering coefficient (Figure 4b) and local efficiency (Figure 4d) in the right orbital mid‐frontal gyrus (ORBmid.R), a critical hub bridging the default mode network (DMN) and salience network, likely impair cross‐network integration efficiency, potentially driving EMCI‐to‐AD progression; as shown in Figure 5c, abnormal decreases in betweenness centrality in the left middle cingulate gyrus (DCG.L) spatially correlate with early‐stage tau deposition patterns, establishing structural‐functional convergence for executive dysfunction in EMCI.

### 3.4 Discussion

Although the group average of global topological metrics of the brain functional networks of the subjects in the EMCI group changed compared with the subjects in the NC group, mainly in the form of a rise in the length of the characteristic paths and a decrease in the global average clustering coefficients and global efficiencies, the statistically significant intergroup differences between the two subject groups were not significant. This suggests that although EMCI patients showed a certain degree of whole‐brain functional impairment, the analysis based on global topological properties does not well demonstrate the variability caused by the impairment, which may be a role played by the compensatory mechanisms existing within the functional brain network. When there is abnormal damage to some parts of the functional connectivity of the brain functional network, the original brain functional network enhances the strength of other parts of the functional connectivity to compensate for the function of the damaged region. The brain may activate normally inactive networks to compensate for the damaged area, the brain may change the connection pattern of its internal networks, enable alternative pathways or activate parallel networks to maintain specific functions, and certain functions may be shifted from damaged brain regions to other undamaged brain regions, thus ensuring the cognitive functioning of the brain as much as possible. This compensatory mechanism of the brain also ensures the restoration of cognitive function through interventions and treatments in the later stages of neurodegenerative diseases.

The possible existence of compensatory mechanisms in the brain functional networks does not indicate statistically significant differences in the global topological properties of the brain functional networks compared with normal subjects, even in patients with early mild cognitive impairment who clinically show initial cognitive decline and memory loss, which makes the global topological properties of the brain functional networks not a clinically specific indicator for distinguishing and screening patients with EMCI indicators.

Based on the whole‐brain network study, we are not well able to uncover the network‐specific pathogenesis of brain function in EMCI patients. For the measurement and study of brain functional connectivity and network topology, in addition to the perspective of the whole brain network, it is also possible to start exploring the nodes of brain regions in the network, so as to obtain a more fine‐grained analysis of the brain functional changes associated with clinical cognitive impairment. Further, compared with exploring the abnormalities in brain functional connections of MCI patients under specific conditions [26], this paper carries out a global brain function and brain region function abnormality study based on the pivotal interruption method on the brain function network of the subjects, and refined the location and characteristics of brain functional abnormalities in EMCI patients [27, 28, 29, 30].

Degree centrality and median centrality of brain regions reflect the information transmission status of brain regions in the brain functional network. Brain regions with high degree centrality or median centrality coefficients tend to act as key hubs for the efficient communication of information in the brain functional network, whereas those with low degree and median centrality coefficients indicate that the brain regions belong to a non‐core and key role in the whole‐brain information transmission and communication. Statistical analysis revealed that the brain regions of the subjects in the EMCI group showed an increase or decrease in degree centrality and median centrality compared with the average of the subjects in the NC group, which suggests that the EMCI patients may have experienced abnormalities in the working mechanism of some brain regions, which may also affect the information transmission and communication among the whole‐brain functional networks.

The clustering coefficients of brain regions reflect the local connectivity patterns between brain regions and neighbouring brain regions, which help brain regions collaborate with each other to process cognitive information and perform cognitive tasks. The clustering coefficients of some of the brain regions of the subjects in the EMCI group were abnormally high and low compared with the average clustering coefficients of the subjects in the NC group, which may reflect the abnormalities or impairments of some of the brain regions of the patients with EMCI in the process of information processing of the whole‐brain functional network. Abnormalities or impairments in information processing in the whole‐brain functional network.

The local efficiency of brain regions is a comprehensive measure of the ability of brain regions to efficiently interact and process information while interconnecting with neighbouring brain region nodes. The abnormal increase and decrease in local efficiency of some brain regions of the subjects in the EMCI group compared with the average local efficiency of the subjects in the NC group also further reflects the possibility that some of the brain regions of the patients with EMCI may have an abnormal mechanism and affect the ability of the whole‐brain function to communicate information with an abnormal Mechanisms.

Through the pivot disruption index analysis of degree centrality, clustering coefficient, meso‐centrality, and local efficiency, this paper found that the local topological properties of some brain regions of EMCI patients were abnormally elevated (brain region active) or reduced (brain region inactive), indicating that these brain regions might have been damaged compared with normal subjects, which in turn affected the stability of the whole brain functional network and cognitive work mechanism. Statistical analysis of the pivotal disruption indices of the two subject groups revealed significant differences, which helped us to understand and explore the abnormal mechanisms of EMCI brain functional networks through a more fine‐grained brain region perspective, and the pivotal disruption indices of the local efficiency of brain region nodes may serve as a clinically specific indicator for effective screening and diagnosis of brain dysfunction in EMCI patients.

By analysing the abnormal brain regions with pivotal interruptions in the functional brain network of EMCI patients, this paper identified nodes in the functional brain network of EMCI subjects with abnormally elevated local topological attributes in some of the brain nodes, such as the left olfactory cortex (OLF.L), the right olfactory cortex (OLF.R), and the right dorsolateral supra frontal gyrus (SFGdor.R); and abnormally lowered topological metrics in some of the brain nodes, such as the abnormal increase or decrease of topological metrics in some brain regions in the functional brain network of the EMCI group also further indicated the existence of compensatory mechanisms in the functional brain network, and the increase of topological metrics in degree centrality, local efficiency, median centrality, and clustering coefficient in some brain regions may compensate for the increase of topological metrics in other brain regions. It may be to compensate for the negative impact on the transmission and processing of information in the whole brain functional network caused by the decrease in the topological properties of nodes in other parts of the brain region, so as to maintain the robustness and efficient working mechanism of the whole brain functional network as much as possible. However, the abnormal elevation of the topological properties of these nodes indicates that they may need to carry too much of the brain's cognitive information processing and communication demands in the whole‐brain functional network, which may lead to overloading of the node's function and affect its long‐term functional stability and efficiency. Over‐reliance on a single node may increase the vulnerability of the network, as once this central node is compromised, the efficiency and functionality of the entire network may suffer significant impacts, which may in turn lead to neurological damage and cognitive decline. Abnormal functional network centrality may alter the way the brain processes cognitive tasks, which has been linked to impairments in attention, memory, decision‐making, and other cognitive functions.

## 4 Conclusion

In this paper, the network properties and abnormal brain regions of the functional brain networks of EMCI and NC subjects were explored and analysed based on the functional brain networks, combining complex network and graph theory analysis methods. Firstly, the global topological properties of the brain functional networks of the subjects were analysed, and based on the independent samples *t*‐test method, no significant difference was found between the two groups in the global topological properties of the two groups; then the local topological properties of the brain area nodes of the brain functional networks of the two groups were analysed in terms of pivotal interruption index, and the degree centrality, local clustering coefficients, median centrality, and local efficiencies of brain area nodes of the brain functional networks of the two groups were presented after statistical analysis. Statistical analysis presented significant differences between the groups of subjects, which indicated that there were some brain regions abnormally active or deactivated in the brain functional network of EMCI patients, followed by further analysis of the brain regions that showed abnormalities and discussion of possible compensatory mechanisms in the brain functional network. Compared with traditional studies that only focus on the overall brain network, this study accurately identifies the abnormal characteristics of EMCI patients at the brain region level, providing new ideas for the mining of early diagnostic markers. In the future, the sample size will be expanded, combined with multimodal imaging data, to further explore the dynamic association between these brain abnormalities and cognitive decline, thereby promoting the transformation of research results into clinical applications.

## Author Contributions

**Dong Qi:** data curation, funding acquisition, supervision. **Weiping Wang:** conceptualization, data curation, funding acquisition, resources, supervision **Yapeng Diao:** investigation, methodology, validation, writing – original draft, writing – review and editing. **Wenxiu Zhao:** conceptualization, investigation, methodology, resources, supervision, validation. **Wenqin Rong:** formal analysis, validation, visualization, writing – original draft **Haiyan Zhao:** conceptualization, project administration, resources, supervision.

## Funding

This work was supported in part by National Natural Science Foundation of China under Grant 62271045, in part by Tianjin Science and Technology Project under Grant 25ZXDFQY00290, in part by the Fundamental Research Funds for the Central Universities under Grant FRF‐KST‐25‐008, in part by the Young Teaching Backbone Talent Program of the University of Science and Technology Beijing under Grant JXGG202509, and the CCF‐NSFOCUS “Kunpeng” Research Fund under Grant CCF‐NSFOCUS 202417. The author is also supported by the 2024 Xiaomi Young Scholar Program.

## Conflicts of Interest

The authors declare no conflicts of interest.

## Data Availability

The raw data supporting the conclusions of this article will be made available by the authors on request.
